# New Instruments for Lenticule Extraction in Small Incision Lenticule Extraction (SMILE)

**DOI:** 10.1371/journal.pone.0113774

**Published:** 2014-12-01

**Authors:** Yu-Chi Liu, Tarak Pujara, Jodhbir S. Mehta

**Affiliations:** 1 Singapore Eye Research Institute, Singapore, Singapore; 2 Singapore National Eye Centre, Singapore, Singapore; 3 Carl Zeiss Meditec AG, Singapore, Singapore; 4 Department of Clinical Sciences, Duke-NUS Graduate Medical School, Singapore, Singapore; Bascom Palmer Eye Institute, University of Miami School of Medicine, United States of America

## Abstract

Small incision lenticule extraction (SMILE) is an alternative to Laser-Assisted in situ Keratomileusis (LASIK) for correction of myopia. In cases where surgeons inadvertently dissect the posterior surface first, identification of the anterior surface and subsequent removal become difficult since the anterior surface of the lenticule is compacted against the anterior stromal surface. This may result in incomplete lenticule removal, and a remnant of intrastromal lenticule in SMILE may lead to visual sequelae. In order to aid surgeons in lenticule removal, we have designed and developed 5 novel SMILE lenticule strippers to locate and extract the lenticules more easily. The aim of this study was to investigate and compare the efficacy and quality of these lenticule strippers in assisting SMILE. Thirty porcine eyes were used. The ease of extraction and removal of the lenticule with different strippers was graded by an experienced SMILE surgeon, the extracted lenticule circularity was evaluated by calculating the lenticule circularity, and the intactness of the extracted lenticule edge was assessed using scanning electron microscopy. We found these novel strippers can be of great help to improve the safety and quality of SMILE surgery, particularly in those cases of difficult lenticule extraction.

## Introduction

During the last 4 years, small incision lenticule extractction (SMILE) has become clinically available in Europe and Asia as an alternative to Laser-Assisted in situ Keratomileusis (LASIK) for correction of myopia [1]. It is currently undergoing FDA approval in the USA. In SMILE, a femtosecond laser is used to create an intrastromal lenticule and a small peripheral incision. Following lenticule creation, removal is ideally performed by blunt separation of the anterior surface of the lenticule followed by the posterior surface. The lenticule is then grasped and removed with forceps. In cases where surgeons inadvertently dissect the posterior surface first, identification of the anterior surface and subsequent removal are difficult since the anterior surface of the lenticule is compacted against the anterior stromal surface. This may result in incomplete lenticule removal. A remnant of intrastromal lenticule may lead to visual sequelae, such as irregular astigmatism, depending on its location [2], [3]. In order to aid the surgeon in lenticule removal, we have designed and developed new SMILE lenticule strippers to locate and extract the lenticule. The aim of this study was to investigate and compare the efficacy and quality of five prototype novel lenticule strippers in assisting SMILE.

## Methods

**Figure 1:**
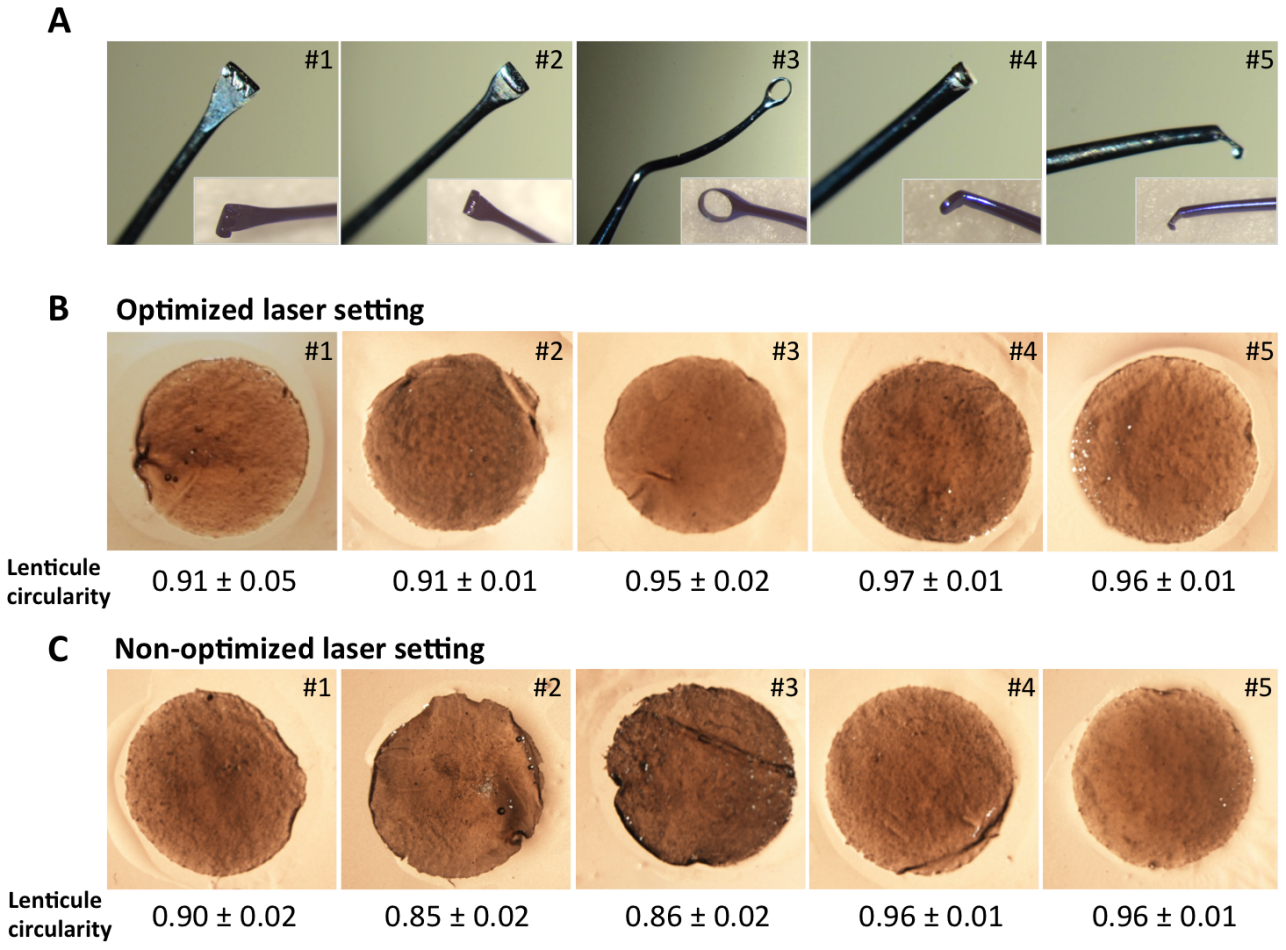
The pictures of the extracted lenticules and the mean lenticule circularity for different instruments. (A) Different designed SMILE lenticule strippers. (B) The corresponding extracted lenticules and the mean lenticule circularity for different instruments in the optimized laser setting. A circularity value approaching 1.0 indicates a circular profile. There was no significant difference in the lenticule circularity between any two of the instruments. (C) The corresponding extracted lenticules and the mean lenticule circularity for different instruments in the non-optimized laser setting, simulating difficult extracting conditions. Torn and distorted edges were observed in the lenticules extracted by No. 2 and No. 3 lenticule strippers, whereas the lenticules extracted by No. 4 or No.5 lenticule strippers maintained almost round shape with smoother and more even edge. The lenticule circularity for No. 2 or No. 3 lenticule strippers were significantly lower than that for No. 4 and No. 5 lenticule strippers.

SMILE procedure was performed on 30 freshly enucleated porcine eyes (post-mortem time <8 hours), which were obtained from a local abattoir (Primary Industries Pte Ltd, Singapore). A myopic correction of −6.00 D was performed with a 500-kHz femtosecond laser (Visumax; Carl Zeiss Meditec) as described previously [4]. Briefly, the eye was docked under the small curved interface cone and suction applied. The femtosecond incisions were performed in a spiral in/out scanning pattern direction [4]. The femtosecond laser parameters were: 110 µm cap thickness, 7.5 mm cap diameter, and 6.5 mm lenticule diameter. The spot distance and tracking spacing were set at 4.5 µm for the cap and lenticule, and at 2.5 µm for the side cuts. Side cut angles were at 90°, incision position at 120°, and incision width 2.5 mm. The surgery was performed with 2 different laser energy settings: an optimized setting using a laser energy of 170 nJ, and a non-optimized comparison using a laser energy of 110 nJ to simulate clinical cases in which the laser settings are not optimized and the lenticule may be more adhesive. After completion of the laser firing, the cornea incision was opened with a Sinskey hook. Identification of the anterior and posterior surface edge of the lenticule was made, and the posterior surface of the lenticule was bluntly dissected on purpose with a Chansue dissector. The refractive lenticule was then extracted from posterior surface with one of the five different investigated SMILE lenticule strippers (Video S1). These lenticule strippers have different designed tips (Figure 1A). Six eyes were used for the experiments of each type of instruments (3 for the optimized setting group, and another 3 for the non-optimized setting group). All the procedures were performed by an experienced SMILE surgeon (JSM). The ease of extraction and removal of the lenticule from the intrastromal space was graded using a modified scoring system reported previously [5], from 1 (poor) to 5 (excellent) (Table 1).

**Table 1:** Subjective grading of extraction/removal of lenticules.

Grading	Extraction and removal of lenticules
1	Severe resistance to extract/remove, resulting in significant torn or distorted lenticule edge
2	Moderate resistance to extract/remove, resulting in noticeable torn or distorted lenticule edge
3	Mild resistance to extract/remove, with limited lenticule edge tearing or distortion
4	Minimal resistance to extract/remove, with mild lenticule edge tearing or distortion
5	No resistance to extract/remove, with no lenticule edge tearing or distortion

Morphometric analysis was also performed. Morphometric data of the area and perimeter of each lenticule was determined by manually outlining point-to-point tracing of lenticule borders using ImageJ software. The lenticule circularity was then calculated using the formula: circularity  = 4π x Area/(perimeter)^2^, where a value approaching 1.0 indicates a circular profile [6]. For each lenticule, the circularity measurement was repeated six times by two independent observers (YCL, LSM), and the average was taken. The edges of the extracted lenticules were then evaluated using scanning electron microscopy (SEM). The sample processing was performed as described previously [7]. For each lenticule, four 75x magnification micrographs in each quadrant, and 300x magnification micrographs in each clock hour, were taken. The remaining stromal tissue after lenticule extraction was also evaluated by SEM.

All data was expressed as mean ± standard deviation. Statistical comparisons among the five instruments were performed using Kruskal–Wallis test with Dunn post-hoc tests. *P* values less than.05 were considered statistically significant.

## Results

The qualitative assessments of the ease of lenticule extraction with different instruments are shown in Table 2. No. 5 lenticule stripper had the best and most satisfactory mean score, and No. 3 lenticule stripper had the worst score of assessments, in both the optimized and non-optimized settings (*P* = 0.007 and *P*<0.001, respectively).

**Table 2:** The qualitative assessments of the ease of lenticule extraction with different instruments.

SMILE lenticule stripper	Mean assessment scores	
Optimized laser setting		Non-optimized laser setting
#1	3.7±0.6	2.3±0.6
#2	3.0±0.0	2.0±0.0
#3	2.0±0.0	1.0±0.0
#4	4.0±0.0	4.0±1.0
#5	4.7±0.6	5.0±0.0

The pictures of the extracted lenticules and the mean lenticule circularity for different instruments are shown in Figure 1. In the optimized laser setting, all the extracted lenticules were round and intact. There was no significant difference in the lenticule circularity between any two of the instruments. In the non-optimized setting, torn and distorted edges were observed in the lenticules extracted by No. 2 and No. 3 lenticule strippers, whereas the lenticules extracted by No. 4 or No.5 maintained almost a round shape with smoother and more even edge. The lenticule circularity for No. 2 or No. 3 lenticule strippers were 0.85±0.02 and 0.86±0.02, which were significantly lower than that for No. 4 and No. 5 lenticule strippers (0.96±0.01 and 0.96±0.01; all *P* values <0.001).

**Figure 2:**
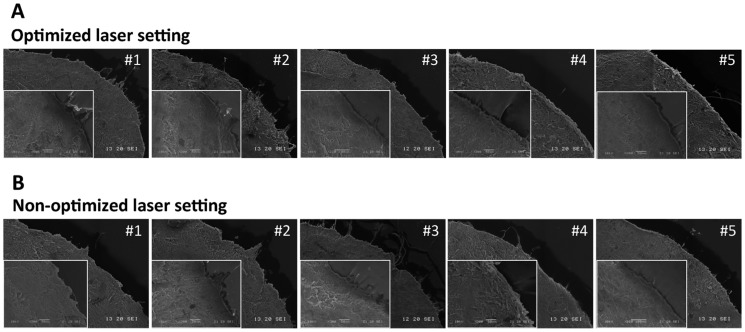
Representative SEM micrographs of the lenitcules extracted by different instruments (center: 75x, scale bar: 200 µm; left corner: 300x, scale bar: 100 µm). (A) The lenticules extracted with the optimized laser setting. (B) The lenticules extracted with non-optimized laser setting. The lenticules extracted by No. 2 or No.3 lenticule strippers had regional torn or distorted edge, with edge tags on. On the contrary, the lenticules extracted by No. 4 or No. 5 lenticule strippers maintained the edge more intact with less edge tags on, either in the optimized or non-optimized laser setting. Magnification: 75x, scale bar: 200 µm.

**Figure 3:**
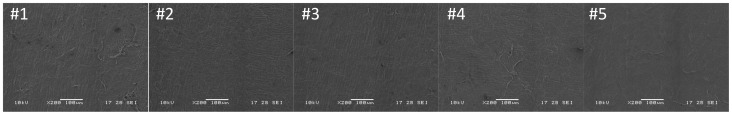
Representative SEM micrographs showing the remaining stroma after the lenitcule extraction by different instruments in the non-optimizing laser setting. The remaining stroma after the lenticule extraction remained intact, without damage caused by the instruments. Magnification 200x, scale bar: 100 µm.

SEM micrographs demonstrated similar results. In both the optimized and non-optimized settings, the lenticules extracted by No. 2 or No.3 lenticule strippers had regional torn or distorted edges, with edge tags on them. On the contrary, the lenticules extracted by No. 4 or No. 5 lenticule strippers maintained the edge more intact with less edge tags (Figure 2). The remaining stroma after the lenticule extraction remained intact, without damage caused by the instruments, even in the non-optimized laser setting (Figure 3).

## Discussion

Surgical maneuvers in SMILE procedure are more challenging than with femtosecond lenticule extraction (FLEx) or pseudoSMILE [8], since they have to be performed through a smaller arcuate incision (2.5–4 mm). This may be associated with increased intraoperative complications [9], [10], especially in lower myopic treatments, with thin lenticules, where delineation of the anterior surface of the lenticule may be unperceived. When the initial dissection undermines the posterior surface of the lenticule, the surgery becomes difficult, as the lenticule sticks to the anterior corneal cap [9]. Hence it is crucial to find the first dissection plane on the anterior surface of the lenticule first to avoid this. Moreover, the edge of the lenticule sometimes can not be visualized easily to be grasped by forceps.

Currently, there are no standard instruments to extract the lenticule in SMILE especially in cases of retained lenticules following improper or difficult dissection. Various types of forceps, such as Tan DSAEK forceps (used in our institute; Figure S1) [11], modified serrated McPherson forceps [9], or Shah forceps [12], are currently used to “grasp” the thin lenticuleInstead of grasping the lenticule using forceps, which may lead to lenticule tears and retention of fragments of the lenticule, we have developed the instruments in this study to prevent these complications. Improper dissection may occur when surgeons inadvertently dissect the posterior plane first, and may also occur when the lenticule is thin, e.g. in low myopic treatment, or when the edge of the lenticule is not easily visible. In these situations, the designed strippers can be simply inserted into the plane between the posterior surface of lenticule and underlying stroma, and then “hook out” the lenticule directly. In the present study, No. 5 lenticule stripper had a significantly higher score in the both optimized and non-optimized laser settings with respect to the ease of lenticule extraction, and we thought this was related to the geometry of the designed tip. The tip of No.5 stripper had a double-hook structure, which worked as a point of the application of force to extract the lenticule. In comparison, the tip of No. 3 stripper was a planer ring, which had no single point of force to exert the lenticule and hence had the lowest score. Similarly, the planer “claw” shape of No. 1 stripper's tip, and the planar “retractor” shape of No. 2 stripper's tip, had more difficulty in exerting a uniform force to extract the lenticule as compared to the “single-hook” shape of No. 4 stripper's tip. The SEM evaluation and circularity assessments demonstrated similar results.

In the present study, the energy level of 170 nJ was used for the optimized laser setting because it is the energy level we use for the clinical SMILE patients in our institute. The energy level for patients in other clinical published studies ranges from 150 nJ to 170 nJ [1], [12]. For the non-optimized setting, we reduced the optimized laser energy by 10% (153 nj), 20% (136 nJ), 30% (119 nJ), 35% (110.5 nj), and 40% (102 nJ) in sequence till we found a setting in which there was no laser cavitation bubbles formation and laser lamellar cutting could not be performed successfully. Therefore, the energy level of 110 nJ (35% reduction) was chosen for the non-optimized laser setting.

In a large study evaluating the safety and complications of more than 1500 SMILE procedures [1], the authors reported difficulties extracting the lenticule in 1.9% of all the procedures, and the subsequent lenticule extraction led to perforation of the cap, or a tear at the incision site. Difficult extraction was also associated with postoperative topographic irregularities, probably due to irregular cleavage of tissue bridges. Therefore, it is important to completely dissect and remove the lenticule within the stroma and check the intactness of the extracted lenticule. Our results demonstrated the lenticules extracted by No. 4 and No. 5 lenticule strippers maintained an intact round shape with no edge tears or distortion confirmed by the SEM experiments and circularity measurements. These two strippers also had high surgeon assessment scores, especially in the non-optimized laser setting, hence would be of great help to improve the safety and quality of SMILE surgery, particularly in those cases of difficult lenticule extraction.

## Supporting Information

Figure S1 The pictures, mean circularity and SEM micrographs of the lenticules extracted by Tan DSAEK forceps (n = 3). (A) The lenticule extracted in the optimized laser setting; the mean circularity was 0.96±0.01. (B) The lenticule extracted in the non-optimized laser setting; the mean circularity was 0.94±0.01. (C) The corresponding SEM micrograph of the lenitcule extracted in the optimized laser setting. (D) The corresponding SEM micrograph of the lenitcule extracted in the non-optimized laser setting. Center: 75x, left corner: 300x.
(TIF)

Video S1 A representative video showing the use of SMILE lenticule strippers to extract refractive lenticules. After completion of the laser firing with the optimized laser setting, the posterior surface of the lenticule was bluntly dissected with a Chansue dissector. The lenticule was then extracted from the posterior surface with a No. 5 lenticule stripper.
(MP4)

## References

[1] Ivarsen A, Asp S, Hjortdal J (2014) Safety and complications of more than 1500 small-incision lenticule extraction procedures. Ophthalmology 121:822–828. 24365175 10.1016/j.ophtha.2013.11.006

[2] Sekundo W, Gertnere J, Bertelmann T, Solomatin I (2014) One-year refractive results, contrast sensitivity, high-order aberrations and complications after myopic small-incision lenticule extraction (ReLEx SMILE). Graefes Arch Clin Exp Ophthalmol 252:837–843. 24647595 10.1007/s00417-014-2608-4

[3] Dong Z, Zhou X (2013) Irregular astigmatism after femtosecond laser refractive lenticule extraction. J Cataract Refract Surg 39:952–954. 23688883 10.1016/j.jcrs.2013.04.016

[4] Riau AK, Ang HP, Lwin NC, Chaurasia SS, Tan DT, et al (2013) Comparison of four different VisuMax circle patterns for flap creation after small incision lenticule extraction. J Refract Surg 29:236–244. 23557221 10.3928/1081597X-20130318-02

[5] Mehta JS, Parthasarthy A, Por YM, Cajucom-Uy H, Beuerman RW, et al (2008) Femtosecond laser-assisted endothelial keratoplasty: a laboratory model. Cornea 27:706–712. 18580264 10.1097/QAI.0b013e31815ee267

[6] Peh GS, Toh KP, Wu FY, Tan DT, Mehta JS (2011) Cultivation of human corneal endothelial cells isolated from paired donor corneas. PLoS One 6:e28310. 22194824 10.1371/journal.pone.0028310 PMC3241625

[7] Riau AK, Liu YC, Lwin NC, Ang HP, Tan NY, et al (2014) Comparative study of nJ- and μJ-energy level femtosecond lasers: Evaluation of flap adhesion strength, stromal bed quality and tissue responses. Invest Ophthalmol Vis Sci 55:3186–3194. 24764066 10.1167/iovs.14-14434

[8] Ang M, Mehta JS, Chan C, Koh JC, Htoon HM, et al (2014) Refractive Lenticule Extraction: Transition and Comparison of Three Surgical Techniques. J Cataract Refract Surg 40:1415–1424. 25135532 10.1016/j.jcrs.2013.12.026

[9] Sekundo W, Kunert KS, Blum M (2011) Small incision corneal refractive surgery using the small incision lenticule extraction (SMILE) procedure for the correction of myopia and myopic astigmatism: results of a 6 month prospective study. Br J Ophthalmol 95:335–339. 20601657 10.1136/bjo.2009.174284

[10] Vestergaard AH, Grauslund J, Ivarsen AR, Hjortdal JØ (2014) Efficacy, safety, predictability, contrast sensitivity, and aberrations after femtosecond laser lenticule extraction. J Cataract Refract Surg 40:403–411. 24480564 10.1016/j.jcrs.2013.07.053

[11] Mohamed-Noriega K, Riau AK, Lwin NC, Chaurasia SS, Tan DT, et al (2014) Early corneal nerve damage and recovery following small incision lenticule extraction (SMILE) and laser in situ keratomileusis (LASIK). Invest Ophthalmol Vis Sci. 25 55:1823–34. 24569584 10.1167/iovs.13-13324

[12] Shah R, Shah S, Sengupta S (2011) Results of small incision lenticule extraction: All-in-one femtosecond laser refractive surgery. J Cataract Refract Surg 37:127–37. 21183108 10.1016/j.jcrs.2010.07.033

