# Supplementary figures and images for: New Instruments for Lenticule Extraction in Small Incision Lenticule Extraction (SMILE)
Source: PLoS One. 2014 Dec 1;9(12):e113774. doi: 10.1371/journal.pone.0113774 (PMC4249972; doi:10.1371/journal.pone.0113774)

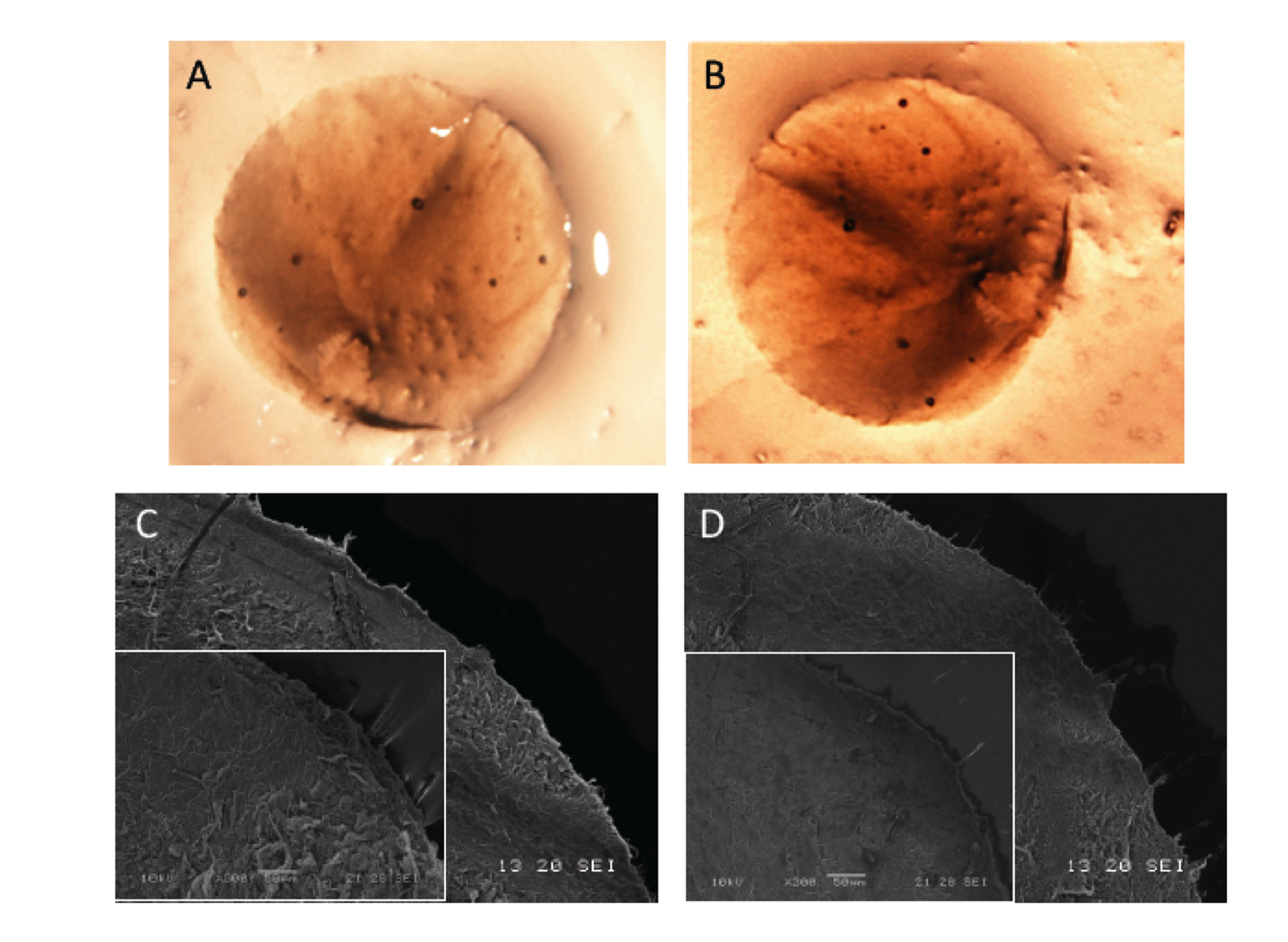

Supplement: Figure S1 — The pictures, mean circularity and SEM micrographs of the lenticules extracted by Tan DSAEK forceps (n = 3). (A) The lenticule extracted in the optimized laser setting; the mean circularity was 0.96±0.01. (B) The lenticule extracted in the non-optimized laser setting; the mean circularity was 0.94±0.01. (C) The corresponding SEM micrograph of the lenitcule extracted in the optimized laser setting. (D) The corresponding SEM micrograph of the lenitcule extracted in the non-optimized laser setting. Center: 75x, left corner: 300x. (TIF) [file pone.0113774.s001.tif]
